# Selection of suitable reference genes for normalization of quantitative RT-PCR in peripheral blood samples of bottlenose dolphins (*Tursiops truncatus*)

**DOI:** 10.1038/srep15425

**Published:** 2015-10-21

**Authors:** I-Hua Chen, Lien-Siang Chou, Shih-Jen Chou, Jiann-Hsiung Wang, Jeffrey Stott, Myra Blanchard, I-Fan Jen, Wei-Cheng Yang

**Affiliations:** 1 Department of Veterinary Medicine, National Chiayi University, Chiayi, 600, Taiwan (R.O.C.); 2 Institute of Ecology and Evolutionary Biology, National Taiwan University, Taipei, 106, Taiwan (R.O.C.); 3 School of Veterinary Medicine, University of California, Davis, California, 95616, United States of America; 4 Farglory Ocean Park, Hualien, 604, Taiwan (R.O.C.)

## Abstract

Quantitative RT-PCR is often used as a research tool directed at gene transcription. Selection of optimal housekeeping genes (HKGs) as reference genes is critical to establishing sensitive and reproducible qRT-PCR-based assays. The current study was designed to identify the appropriate reference genes in blood leukocytes of bottlenose dolphins (*Tursiops truncatus*) for gene transcription research. Seventy-five blood samples collected from 7 bottlenose dolphins were used to analyze 15 candidate HKGs (ACTB, B2M, GAPDH, HPRT1, LDHB, PGK1, RPL4, RPL8, RPL18, RPS9, RPS18, TFRC, YWHAZ, LDHA, SDHA). HKG stability in qRT-PCR was determined using geNorm, NormFinder, BestKeeper and comparative delta Ct algorithms. Utilization of RefFinder, which combined all 4 algorithms, suggested that PGK1, HPRT1 and RPL4 were the most stable HKGs in bottlenose dolphin blood. Gene transcription perturbations in blood can serve as an indication of health status in cetaceans as it occurs prior to alterations in hematology and chemistry. This study identified HKGs that could be used in gene transcript studies, which may contribute to further mRNA relative quantification research in the peripheral blood leukocytes in captive cetaceans.

Quantitative reverse transcription PCR (qRT-PCR) represents a rapid and reliable method for the detection and quantification of mRNA transcripts of a selected gene of interest (GOI) and is well suited to study biological processes, many of which can have practical clinical applications^1^. When only a small number of cells are available and the number of GOIs is limited, qRT-PCR provides the simultaneous measurement of gene transcripts in many different samples^2^. Furthermore, this method becomes the only technique that can detect small number of mRNA copies since expression level for some genes is often low^1^. Such an approach is especially attractive for measuring gene transcript levels in species for which reagents for protein detection and bioassay are not readily available. Relative quantification in qRT-PCR can determine the transcript level change in given samples relative to another control sample. This technique is dependent upon the use of an internal control gene for normalization, which controls multiple variables such as RNA integrity, cDNA concentration, enzymatic efficiencies, and transcriptional activity differences between tissues^3^. The use of appropriate controls in the data normalization step is essential for accurate comparison of mRNA measurements between different samples^4^.

Housekeeping genes (HKGs), which are involved in basic metabolism and maintenance of the cell, are commonly recognized as reference genes used to normalize qRT-PCR. An ideal reference gene should present stable transcript levels when exposed to the same experimental protocol of the GOI. However, some of the most commonly used HKGs display significantly different transcript levels in various tissues and cannot always serve as reliable controls (reviewed in^5^,^6^). Thus, conducting preliminary evaluations for identifying stable HKGs for a qRT-PCR assay in a given species and tissue is desirable.

Cetacean species share key immunologic components with those previously defined in lab animals and humans^7^. Recent years have seen increased attention being given to cetacean immunology due to the important value of the animals under human care and the significant susceptibility to environmental degradation in free-ranging species serving as sentinels of ecosystem health^8^. Immune gene transcript assessment has potential to be an important approach to evaluate cetacean health. Cetacean gene transcripts have been the subject of quantitative analysis in multiple tissues and species. These studies have been based upon qRT-PCR using a variety of different HKGs, including ribosomal protein L8 (RPL8) in killer whale (*Orcinus orca*) skin biopsies^9^, glyceraldehyde 3-phosphate dehydrogenase (GAPDH) and tyrosin 3-monooxygenase/tryptophan 5-monooxygenase activation protein zeta (YWHAZ) in harbor porpoise (*Phocoena phocoena*) blood samples^10^,^11^,^12^, GAPDH in bottlenose dolphin (*Tursiops truncatus*) blood samples^13^, and RPS9 in blood samples in bottlenose dolphins, beluga whales (*Delphinapterus leucas*), and Pacific white-sided dolphins (*Lagenorhynchus obliquidens*)^8^,^14^. These HKGs were selected based upon their supposedly equal transcript levels in certain tissues derived from different sample groups. Alterations in reference gene transcript levels induced by varying experimental conditions or other factors have the potential to compromise detection of small perturbations resulting in poor sensitivity^15^. Comprehensive evaluations of cetacean reference genes are limited. Spinsanti *et al.*^5^ tested 10 HKGs for the selection of reference genes in qRT-PCR studies directed at striped dolphin (*Stenella coeruleoalba*) skin biopsies, and determined that YWHAZ and GAPDH were superior reference genes. The same 10 HKGs were tested in transcript analysis of striped dolphin fibroblast cultures exposed to organochlorines (OCs), polybrominated diphenyl ethers (PBDEs) and 17β-estradiol^16^. Data from that study suggested that three different pairs of HKGs should be used to normalize qRT-PCR data: YWHAZ and RPS18 for 17β-estradiol, succinate dehydrogenase complex subunit A (SDHA) and YWHAZ for OCs, and GAPDH and YWHAZ for PBDEs. Another reference gene transcript study employing skin biopsies from blue whale (*Balaenoptera musculus*), fin whales (*Balaenoptera physalus*) and sperm whales (*Physeter macrocephalus*) demonstrated HKGs encoding RPL4 and RPS18 were the most suitable controls followed by genes encoding phosphoglycerate kinase (PGK1) and SDHA^17^. Such studies demonstrated the importance of preliminary selection of optimal HKGs for studies with different purposes and target tissues.

This project investigated the suitability of 15 HKGs for normalization of qRT-PCR data derived from whole blood samples of bottlenose dolphin, which is one of the most commonly displayed cetacean species in aquaria worldwide. Besides 12 HKGs have been evaluated or used in previous studies^5^,^8^,^9^,^10^,^11^,^12^,^13^,^14^,^16^,^17^, we also included the other 3 genes that could participate in other different cell functions^18^,^19^ (Table 1). Acquisition of such data is central to our long-term goal of applying qRT-PCR in multidisciplinary immune assessments of captive and free-ranging cetacean species.

## Results

### Amplification efficiency and transcript levels of candidate HKGs

**Figure 1:**
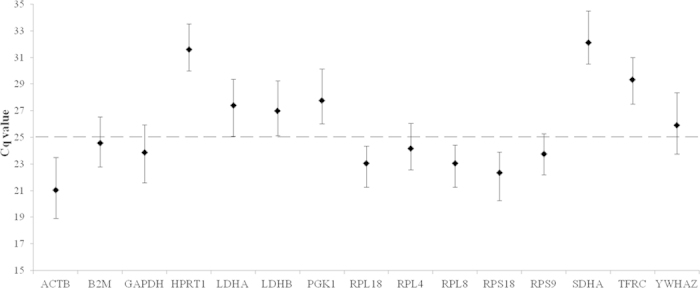
Transcript levels of candidate HKGs derived from blood samples of bottlenose dolphins (*Tursiops truncatus*) (n = 75). Values are given as qPCR cycle threshold numbers (Cq values). Dots represent mean Cq values and whiskers the range of Cq values in the 75 samples.

Amplification efficiency (E) values for the 15 candidate HKGs ranged from 95.19% and 102.29% with R^2^ values being >0.99 (Table 2); therefore all 15 HKGs were included in the analysis of reference gene suitability. Figure 1 illustrated the variable transcript levels in the 15 HKGs with the lowest mean Cq values (21.06) in ACTB, and the highest (32.14) in SDHA. Transcript levels were used to establish two arbitrary categories: those that were highly transcribed (mean Cq values <25 cycles) including ACTB, B2M, GAPDH, RPL4, RPL8, RPL18, RPS18, and RPS9, and those with lower transcript levels (mean Cq values >25 cycles) including HPRT1, LDHA, LDHB, PGK1, SDHA, TFRC and YWHAZ. All HKGs showed a small difference (<5 cycles) between the maximum and minimum Cq values.

### Gene transcript stability in 75-sample group

**Figure 2:**
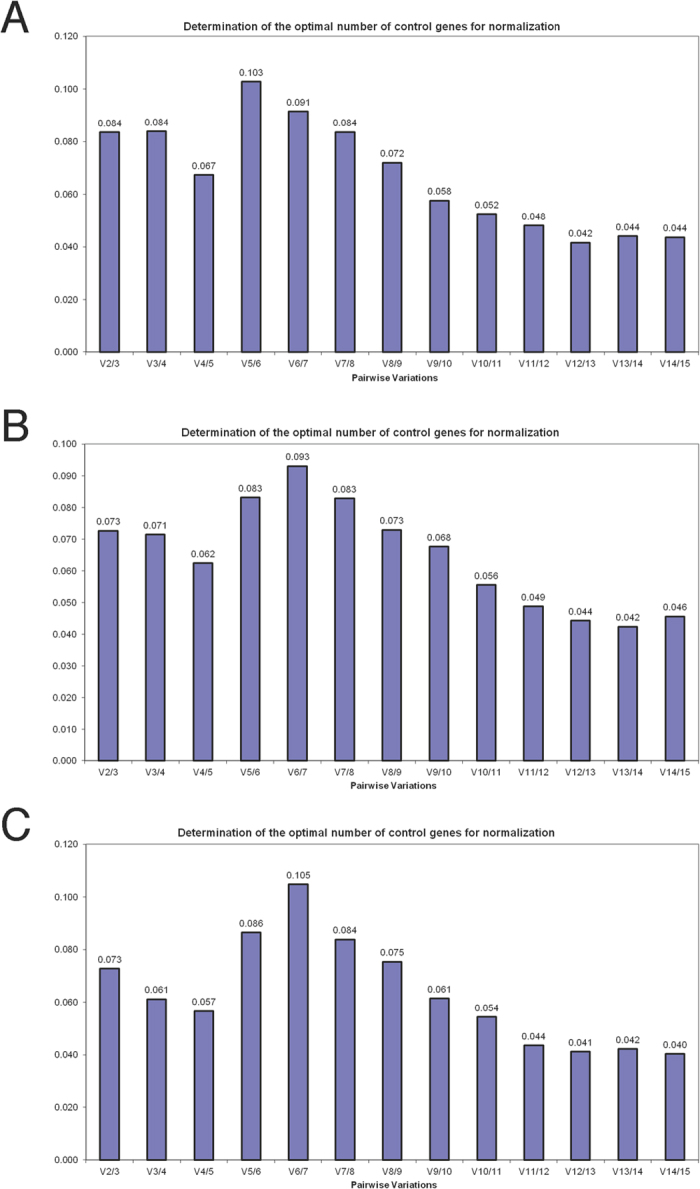
Pairwise variations generated by geNorm algorithm. (**A**) 75 samples; (**B**) 55 samples; (**C**) 35 samples.

**Figure 3:**
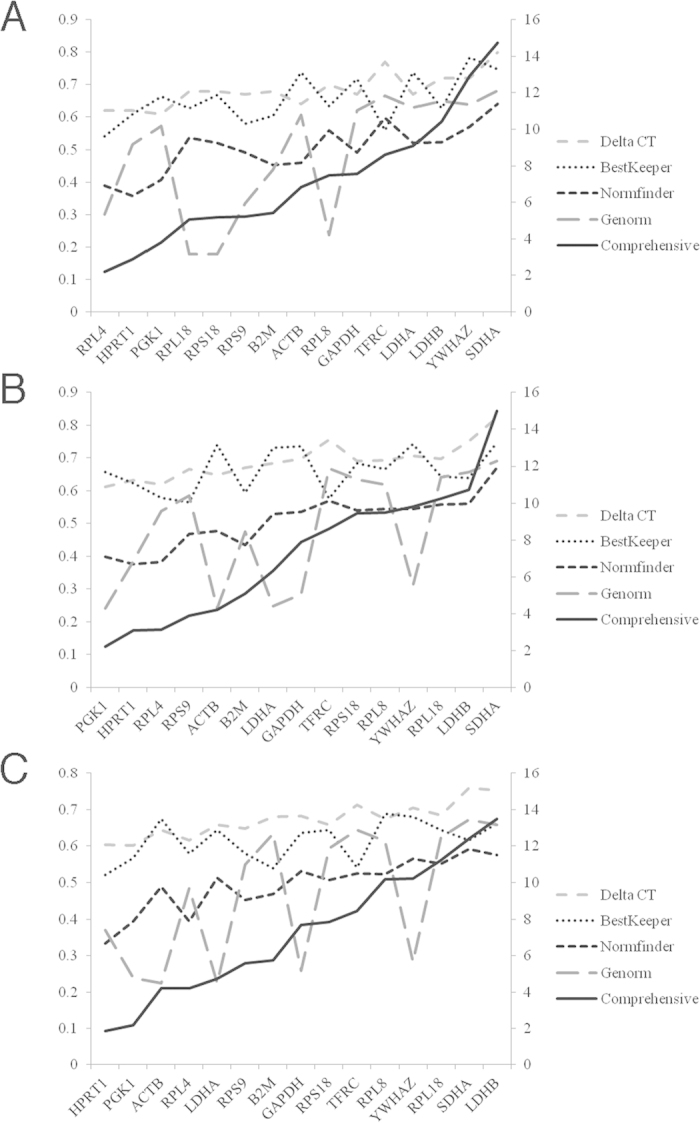
Stability values and ranking orders determined by 4 algorithms and RefFinder. (**A**) 75 samples; (**B**) 55 samples; (**C**) 35 samples.

The geNorm algorithm demonstrated good stability of all tested HKGs (Table 3). M values, average pair-wise variation of a given gene with all other control genes, were lower (0.178–0.682) than the program’s default limit of 1.5. RPL18 and RPS18 were the most stable genes followed by RPL8, RPL4, and RPS9. The optimal number of reference genes for normalization was established using pairwise variation values (V) between two sequential normalization factors while employing an increasing number of genes. The optimal number was determined to be less than 3 based upon V_2/3_ value of 0.084 (<0.15 is the default cut-off) (Fig. 2A), indicating that the addition of one more HKG would not significantly improve reliability. NormFinder analysis identified HPRT1 as having the best stability (0.357) followed by RPL4 (0.390) and PGK1 (0.408) (Table 3). BestKeeper analysis determined the SD_Cq value_ of all HKGs (0.540–0.783) were <1 indicating these genes were basically stably expressed. The three most stable genes, according to their SD_Cq value_,were RPL4, TFRC and RPS9 (Table 3). The best choice in comparative ΔCt method was PGK1, HPRT1 and RPL4. RefFinder identified RPL4, HPRT1 and PGK1 as being superior (Table 3, Fig. 3A).

### Gene transcript stability in 35- and 55-sample groups

In both 35- and 55-sample groups the M values in geNorm of all studied HKGs were lower than the program’s default limit (M = 1.5), and the SD_Cq value_ in BestKeeper were <1. V_2/3_ values in both groups were below to 0.15 (Fig. 2B,C). In contrast to the result in 75-sample group, geNorm algorithm identified PGK1, ACTB and LDHA to be the most stable HKGs in both groups. BestKeeper showed similar results in high-ranking HKGs (RPL4, TFRC, RPS9, B2M and HPRT1) in three groups. NormFinder and comparative ΔCt method identified HPRT1, PGK1 and RPL4 to be the best-three HKGs in all three groups. The RefFinder comprehensive rankings placed PGK1, HPRT1 and RPL4 as being highly ranked HKGs in all three groups (Tables 3, 4, 5, Fig. 3).

## Discussion

RNA transcript stability plays a crucial role in qRT-PCR-based studies due to pre-analytical variations, especially degradation by endogenous RNases and unintentional transcription of individual genes after blood drawing. Without proper preservation, copy number of individual mRNA transcripts in blood can change more than 1000-fold during storage and transport^20^. Two common methods for stabilizing blood leukocyte RNA are to use PAXgene Blood RNA vacutainer tube and RNA*later*. Previous study compared RNA transcript stability in blood leukocytes collected directly into PAXgene tubes or transferred immediately in RNA*later*, and showed both methods were clearly appropriate for RNA stabilization in blood based on good quantity, integrity and purity of isolated RNA^21^. PAXgene tube has been successfully used in blood collection of sea otters (*Enhydra lutris*) to measure differential transcript levels of select immune function genes^20^. PAXgene tubes have the advantage of minimal sample manipulation and immediate exposure of the cells to RNA stabilizing agents, but require collection of 2.5 mL of blood. The present study used RNA*later*. Smaller blood volume (0.5 mL) needed for RNA*later* would facilitate sample collection and transport. Besides, a small difference between the maximum and minimum Cq values of each HKG indicates good quality of RNA stabilization and the consequent procedures in the current study.

Amplification efficiency is an important factor in gene transcript studies using qPCR. When efficiency is not close to 100% (doubling of PCR products per cycle), the calculation the gene quantification requires corrected^22^. ΔΔCt and ΔΔCt_corrected_ may yield similar results only in the case where high quality data are available and the traditional ΔΔCt method would not overestimate the error^23^. The efficiency values of 15 candidate genes in the present study were within the optimal range of 95–105%. Regardless, HKG rankings were susceptible to modest variation if raw Cq values were used for stability analysis rather than corrected Cq values were used (data not shown). This suggests that proper efficiency adjustment can improve qPCR data analysis with greater accuracy.

The HKG stability orders proposed by the four different algorithms used in the current study were not identical, which has been described before^24^. BestKeeper uses raw Cq data as compared to relative transcript levels used in geNorm and NormFinder that may lead to the different outputs^24^. Comparative ΔCt and geNorm, which use a pairwise comparison approach, are prone to select co-regulated genes and this can also influence the ranking results^25^. While NormFinder uses a model-based approach that considers systematic differences and is less likely to be impacted by co-regulated HKGs, it is sensitive to sampling errors and outliers^26^. Since different algorithms can show various HKG rankings, it has been suggested that more than one type of algorithm should be used for reference gene selection^27^. RefFinder was used in the current study to combine all four algorithms to comprehensively evaluate and rank HKGs. This approach assigns an appropriate score to each individual HKG and then calculates their geometric means to produce a final ranking.

The three most stable HKGs (PGK1, RPL4, HPRT1) identified using RefFinder were also in high-ranking orders in NormFinder and comparative ΔCt. In contrast, the top 5 reference genes identified by geNorm were all coding for ribosomal proteins that are likely to be co-regulated. It has been demonstrated that the sensitivity to co-regulation is a major weakness of the pairwise comparison approach while the co-regulation of candidate HKGs does not significantly affect the model-based approach (NormFinder)^26^. Sole utilization of ribosomal protein genes as reference genes has the potential to decrease the sensitivity of identifying changes in transcript levels of GOI in an experiment^6^. Therefore, utilization of HKGs whose encoded proteins belong to different functional classes would reduce the co-regulation effect^26^. The three most stable HKGs in the present study are responsible for different functions. PGK1, encoding for a key enzyme in glycolysis and gluconeogenesis, has previously been identified as a stable reference gene for use with human whole blood RNA and RNA derived from PBMC^28^. RPL4 encodes a protein that is a component of the 60S ribosome subunit. It has been identified as a suitable reference gene on the PBMCs with unknown pathogenic condition in pigs^29^. RPL4 and PGK1 have previously been recommended as reference gene for exfoliated cervical cells^30^. HPRT1, plays a central role in the generation of purine nucleotides through the purine salvage pathway, belonged to one of the most stable reference genes for qRT-PCR studies in human neutrophils^31^ and exercise induced stress in horse PBMCs^32^.

Increasing the number of stably transcribed HKGs included in calculation will increase the efficacy of the normalization factor^3^. Previous studies have suggested there is no single reference gene that can be used for different experiments but rather a group of putative reference genes should be considered for certain specific experimental setups^27^. While inclusion of more HKGs further decreased the V values in the present study, the V2/3 value showed two genes were sufficient for data normalization. Previous study has suggested the transcript levels of a reference gene should not to be very low (Cq > 30) or very high (Cq < 15)^33^. However, appropriate reference genes were suggested to have the same transcript levels as the target gene in an experimental application in order to enhance the uniformity of the analysis^5^. According to mean Cq values, PGK1 and HPRT1 were classified in the low transcript-level group (mean Cq > 25) and RPL4 in the high transcript-level group (mean Cq < 25). Based upon these concepts, the low-level transcripts encoding PGK1 and HPRT1 would be logical reference genes for studying immune-inducible genes with typical low transcript level, and the combination of RPL4 and PGK1 would be more appropriate for higher transcript-level studies. Investigators must recognize that the proposed reference genes in this study would be suitable only when RNA is extracted from RNA*later*-preserved whole blood samples of bottlenose dolphins. It has been shown that some HKGs found to be invariant in proliferating PBMC cultures were unsuitable when studied in whole blood^28^,^34^. For blood samples from other cetacean species, more studies are needed to identify if PGK1, HPRT1 and RPL4 are superior reference genes as well.

Immunologic studies of cetaceans with cytokine gene transcripts have been conducted on blood samples using several different HKGs as reference genes. GAPDH and YWHAZ were used in harbor porpoise studies^10^,^11^,^12^, GAPDH in bottlenose dolphins^13^, and RPS9 in bottlenose dolphins, beluga whales, and Pacific white-sided dolphins^8^,^14^. RPS9 could potentially be a better reference gene than GAPDH and YWHAZ in studies using bottlenose blood samples since it was ranked much higher than the other two genes in the current study.

The reliability of reference gene selection could be affected by sample size. The general recommendation for selecting reference genes using NormFinder is a minimum of 8 samples and 5–10 genes^26^, and GeNorm proposed the use of 8 reference genes and 10 samples^3^. Previous studies directed at reference gene selection in cetaceans have included 30 skin biopsy samples in striped dolphins^5^, and 20 skin biopsy samples from 7 blue whales (*Balaenoptera musculus*), 7 fin whales (*Balaenoptera physalus*) and 6 sperm whales (*Physeter macrocephalus*)^17^. Bovine^35^ and sheep^36^ studies have employed 22 and 16 neutrophil samples, respectively, for use as reference gene selection. The current study employed 75 blood samples from 7 bottlenose dolphins including clinically healthy controls and individuals with a variety of different body conditions, which has been suggested for facilitating optimal reference gene selection for a wide-range of whole blood transcript studies^37^. In addition, the analyses were conducted using randomly selected subsets of 35 and 55 samples for comparative purposes. Analyses of the 35- and 55-sample subsets using RefFinder also identified HPRT1, PGK1 and RPL4 as being the high-ranking genes, only differing in the ranking order. It indicated that a 35-bottlenose dolphin blood sample set with various body conditions could establish reliable HKGs as reference genes. This is the first comparison of sample size effect on reference gene selection to our knowledge. It should be noted that the reference gene identified here is for use in clinical bottlenose dolphin testing. For non-clinical dolphin research, the potential reference gene should be verified first for each experimental condition. Employing a similar approach in other cetacean species in the future would be both time and cost saving.

## Methods

### Sample collection and preservation

The voluntary blood collection of dolphins was performed in accordance with international guidelines, and the protocol has been reviewed and approved by Council of Agriculture of Taiwan (Approval number 1020727724). Seventy-five samples from 7 bottlenose dolphins (13 samples from A animal, 12 from B animal, and 10 from each of the other 5 animals) in Farglory Ocean Park were obtained on a monthly basis or during occasional examinations from 2011 to 2013. Samples were from dolphins with various body conditions including clinically healthy condition (25 samples from 6 animals), inflammation (12 samples from 4 animals), skin lesions (4 samples from 4 animals), and internal diseases with various abnormalities in blood work and cytology (33 samples from 7 animals). Five hundred microliters of EDTA-anticoagulated whole blood was fixed by 1.3 mL RNA*later*^*®*^(Ambion, Applied Biosystems, Foster City, CA, USA) within 5 min after blood collection. Samples were stored at 4 °C in the first 24 h, and then moved to −20 °C for long-term storage.

### RNA extraction and cDNA synthesis

Total RNA was extracted from blood samples using RiboPure™-Blood Kit (Ambion) according to the manufacturer’s instructions. RNA Armor^TM^ Reagent (Protech, Taipei, Taiwan) was added to RNA solutions to eliminate contaminated RNase. RNA integrity was monitored routinely using denaturing gel electrophoresis. RNA concentration was determined using fluorescence-based quantitation method (Qubit™ fluorometer with a Quant-iT™ RNA Assay Kit (Invitrogen, Carlsbad, CA, USA)). RNA samples were adjusted to a concentration of 100 ng prior to analysis. RNA samples were treated with genomic DNA (gDNA) wipeout solution (Qiagen, Valencia, CA, USA). Treated samples were tested by qPCR to ensure the absence of residue gDNA prior to addition of reverse transcription working solution. QuantiTect^®^ Reverse Transcription kit (Qiagen), provided a blend of oligo-dT and random primers, was used for cDNA synthesis. Complementary DNA and unused extracted RNA were stored at −80 °C.

### Primer and probe design

Sequences of the 15 candidate cetacean HKGs (ACTB, B2M, GAPDH, HPRT1, LDHB, PGK1, RPL4, RPL18, RPS9, RPS18, TFRC, YWHAZ, RPL8, SDHA, LDHA) were obtained from GenBank (Table 2). The majority of sequences were obtained from bottlenose dolphin and striped dolphin, while a few were based upon beluga whale, killer whale and fin whale (*Balaenoptera physalus*). Primers and corresponding UPL probes were designed using Roche UPL design software (ProbeFinder, v.2.49) (Table 2). Primer specificity of the 15 candidate genes was confirmed by PCR using Fast-Run Hotstart PCR kit (Protech) and electrophoresis.

### Quantitative PCR

Quantitative PCR was conducted in 48-well reaction plates using the Eco Real-Time PCR System (Illumina, San Diego, CA, USA). Reactions were prepared in a total volume of 10 μL containing 3 μL of 12-fold-diluted cDNA, 0.4 μL of each 10 μM primer, 0.2 μL of UPL probe (Roche, Pleasanton, CA, USA), 5 μL FastStart Essential DNA Probes Master (Roche) and 1 μL of RNase/DNase-free sterile water (Protech). The thermocycle conditions were set as follows: polymerase activation at 95 °C for 10 min, followed by 45 cycles of denaturation at 95 °C for 10 s and combined primer annealing/elongation at 60 °C for 30 s. All reactions including no template controls (NTC) and plate controls were conducted in triplicate. Plate controls contain the same reaction components on every plate. Cq data was analyzed with EcoStudy software (Illumina). A consistent Cq value across plates was obtained allowing the data consolidation from multiple plates into a single study data set. Baseline values were automatically determined for all plates using Eco Software V4.0. Thresholds for each HKG were determined manually (Table 2). Triplicate Cq values with standard deviation (SD) <0.5 were averaged as raw Cq values. PCR amplification efficiency (E) and R^2^ for each probe and primer pair were calculated from the slope of a standard curve using the following equation: E = (10^(−1/slope)^−1) × 100%. The average of at least three E values for each HKG was used as a gene-specific E for following relative quantity transformation. This study was conducted according to MIQE (Minimum information for publication of quantitative real-time PCR experiments) guidelines^38^.

### Data analysis

Corrected Cq values (Cq corr) were transformed from raw Cq values using ΔCq formula, Cq corr = Cq_min_−log_2_ E ^−ΔCq^, modified from Fu *et al.*^39^, where ΔCq is the Cq value of a certain sample minus the Cq value of the sample with the highest transcript level (lowest Cq, Cq_min_) of each HKG. Stability of all HKGs were evaluated and ranked using algorithms geNorm^3^, NormFinder^26^, comparative ΔCt method^40^ and Bestkeeper^41^ using the web-based analysis tool RefFinder (http://www.leonxie.com/referencegene.php)^42^. Algorithm geNorm calculates the expression stability value for each gene and them performs a pair-wise comparison of this gene with the others. NormFinder ranks the set of candidate reference genes according to the least of their estimated variations. Comparative ΔCt method compares relative transcription of pairs of genes and the stability of candidate reference genes is ranked according to repeatability among all samples. BestKeeper determines the standard deviation and the genes are rated based upon variability. RefFinder calculated the geometric mean based upon rankings obtained from each algorithm and provides the final comprehensive ranking. Thirty-five and 55 samples were randomly selected from the original 75 samples, and the HKG ranking results were compared among 35-, 55- and 75-sample groups.

## Additional Information

**How to cite this article**: Chen, I.-H. *et al.* Selection of suitable reference genes for normalization of quantitative RT-PCR in peripheral blood samples of bottlenose dolphins (*Tursiops truncatus*). *Sci. Rep.* **5**, 15425; doi: 10.1038/srep15425 (2015).

## Figures and Tables

**Table 1:** Function, symbol and name of HKGs in this study.

Function	Gene	Name
Carbohydrate	GAPDH	Glyceraldehyde-3-phosphate dehydrogenase
Metabolism	PGK1	Phosphoglycerate kinase 1
	LDHA	Lactate dehydrogenase A
	LDHB	Lactate dehydrogenase B
Ribosomal Protein	RPS9	Ribosomal protein S9
	RPL4	Ribosomal protein L4
	RPL8	Ribosomal protein L8
	RPL18	Ribosomal protein L18
	RPS18	Ribosomal protein S18
MHC	B2M	β-2-microglobin
Transporter	TFRC	Transferrin receptor
Cytoskeleton	ACTB	β-actin
Citric Acid Cycle	SDHA	Succinate dehydrogenase subunit A
Signal	YWHAZ	Tyrosine 3-monooxygenase/tryptophan 5-monooxygenase activation protein zeta
Others	HPRT1	Hypoxantine phosphoribosyltransferase 1

**Table 2:** Name, accession number, primer sequence, probe number, amplicon size, efficiency and R^2^ of 15 candidate HKGs.

HKG Name	Accession Number	Primer Sequence (5′-3′)	UPL Probe Number	Amplicon Size (bp)	Threshold	Efficiency (%) ± SD	R^2^
ACTB	AB603937.1	F-AGGACCTCTATGCCAACACG	157	75	0.020	97.97 ± 0.668	1.000
		R-CCTTCTGCATCCTGTCAGC					
B2M	DQ404542.1	F-GGTGGAGCAATCAGACCTGT	93	78	0.035	95.19 ± 0.056	0.998
		R-GCGTTGGGAGTGAACTCAG					
GAPDH	DQ404538.1	F-CACCTCAAGATCGTCAGCAA	119	81	0.020	97.73 ± 0.186	0.999
		R-GCCGAAGTGGTCATGGAT					
HPRT1	DQ533610.1	F-GTGGCCCTCTGTGTGCTC	120	81	0.012	96.95 ± 1.441	0.996
		R-ACTATTTCTGTTCAGTGCTTTGATGT					
LDHA	AB477023.1	F-TCCACCATGATTAAGGGTTTG	123	97	0.020	97.70 ± 1.782	0.999
		R-CTTTCACAACATCTGAGATTCCA					
LDHB	AB477024.1	F-TCGGGGGTTAACCAGTGTT	161	78	0.005	99.94 ± 1.757	0.993
		R-AGGGTGTCTGCACTTTTCTTG					
PGK1	DQ533611.1	F-CACTGTGGCCTCTGGCATA	108	84	0.015	97.41 ± 0.824	0.999
		R-GCAACAGCCTCAGCATACTTC					
RPL4	DQ404536.1	F-CAGCGCTGGTCATGTCTAAA	119	108	0.035	97.24 ± 0.831	1.000
		R-GCAAAACAGCCTCCTTGGT					
RPL8	GQ141092.1	F-CCATGAATCCTGTGGAGCAT	131	65	0.020	102.29 ± 2.102	1.000
		R-GGTAGAGGGTTTGCCGATG					
RPL18	DQ403041.1	F-GCAAGATCCTCACCTTCGAC	93	104	0.020	98.04 ± 1.608	0.999
		R-GAAATGCCTGTACACCTCTCG					
RPS9	EU638307.1	F-CTGACGCTGGATGAGAAAGAC	155	77	0.020	99.43 ± 0.918	1.000
		R-ACCCCGATACGGACGAGT					
RPS18	DQ404537	F-GTACGAGGCCAGCACACC	114	90	0.020	98.98 ± 0.493	0.999
		R-TAACAGACAACGCCCACAAA					
SDHA	DQ404540.1	F-CGTATCCCGCTCCATGAC	144	73	0.012	101.70 ± 3.729	0.994
		R-CAGGTACACGTGATCCTTCTCA					
TFRC	DQ404541.1	F- TTTAAACCCAGCAGGAGCAT	140	69	0.020	95.36 ± 0.172	0.999
		R- AGTGGCACCAATAGCTCCAA					
YWHAZ	DQ404539	F-TCTCTTGCAAAAACGGCATT	135	76	0.003	99.92 ± 2.681	0.995
		R-TGCTGTCTTTGTATGACTCTTCACT					

**Table 3:** Results of stability among 15 candidate genes computed by 4 algorithms using 75 bottlenose dolphin blood samples.

HKGs	Comprehensive Ranking		Delta CT		BestKeeper		NormFinder		geNorm	
	Geomean of Ranking Value	Rank	Average of SD	Rank	SD	Rank	Stability value	Rank	M value	Rank
RPL4	2.21	1	0.621	3	0.540	1	0.390	2	0.300	4
HPRT1	2.89	2	0.616	2	0.608	5	0.357	1	0.515	7
PGK1	3.83	3	0.612	1	0.664	9	0.408	3	0.572	8
RPL18	5.07	4	0.682	10	0.626	6	0.536	11	0.178	1
RPS18	5.18	5	0.676	8	0.667	10	0.520	9	0.178	1
RPS9	5.21	6	0.675	7	0.579	3	0.492	7	0.334	5
B2M	5.42	7	0.678	9	0.604	4	0.452	4	0.440	6
ACTB	6.82	8	0.636	4	0.738	12	0.460	5	0.606	9
RPL8	7.50	9	0.700	11	0.635	8	0.558	12	0.237	3
GAPDH	7.58	10	0.668	5	0.717	11	0.490	6	0.621	10
TFRC	8.61	11	0.766	14	0.558	2	0.597	14	0.665	14
LDHA	9.10	12	0.674	6	0.739	13	0.520	8	0.630	11
LDHB	10.43	13	0.722	13	0.628	7	0.522	10	0.649	13
YWHAZ	12.94	14	0.715	12	0.783	15	0.571	13	0.638	12
SDHA	14.74	15	0.796	15	0.748	14	0.641	15	0.682	15

**Table 4:** Results of stability among 15 candidate genes computed by 4 algorithms using 55 bottlenose dolphin blood samples.

HKGs	Comprehensive Ranking		Delta CT		BestKeeper		NormFinder		geNorm	
	Geomean of Ranking Value	Rank	Average of SD	Rank	SD	Rank	Stability value	Rank	M value	Rank
PGK1	2.21	1	0.612	1	0.657	8	0.398	3	0.242	1
HPRT1	3.08	2	0.633	3	0.624	5	0.376	1	0.386	6
RPL4	3.13	3	0.619	2	0.578	3	0.382	2	0.539	8
RPS9	3.87	4	0.665	5	0.565	1	0.469	5	0.585	9
ACTB	4.20	5	0.649	4	0.740	13	0.477	6	0.242	1
B2M	5.09	6	0.671	6	0.593	4	0.435	4	0.474	7
LDHA	6.34	7	0.685	7	0.732	11	0.529	7	0.247	3
GAPDH	7.87	8	0.698	10	0.736	12	0.535	8	0.283	4
TFRC	8.61	9	0.755	14	0.577	2	0.570	14	0.669	14
RPS18	9.43	10	0.691	8	0.685	10	0.540	9	0.635	11
RPL8	9.49	11	0.694	9	0.665	9	0.544	10	0.619	10
YWHAZ	9.80	12	0.706	12	0.743	14	0.544	11	0.312	5
RPL18	10.26	13	0.698	11	0.644	7	0.559	12	0.642	12
LDHB	10.72	14	0.751	13	0.638	6	0.561	13	0.656	13
SDHA	15.00	15	0.824	15	0.747	15	0.671	15	0.690	15

**Table 5:** Results of stability among 15 candidate genes computed by 4 algorithms using 35 bottlenose dolphin blood samples.

HKGs	Comprehensive Ranking		Delta CT		BestKeeper		NormFinder		geNorm	
	Geomean of Ranking Value	Rank	Average of SD	Rank	SD	Rank	Stability value	Rank	M value	Rank
HPRT1	1.86	1	0.603	2	0.522	1	0.334	1	0.371	6
PGK1	2.21	2	0.601	1	0.567	4	0.395	2	0.238	3
ACTB	4.20	3	0.644	4	0.675	13	0.489	6	0.225	1
RPL4	4.21	4	0.616	3	0.580	5	0.395	3	0.485	7
LDHA	4.74	5	0.659	7	0.644	9	0.513	8	0.225	1
RPS9	5.57	6	0.648	5	0.580	6	0.453	4	0.549	8
B2M	5.73	7	0.680	9	0.540	2	0.468	5	0.635	12
GAPDH	7.70	8	0.682	10	0.637	8	0.532	11	0.259	4
RPS18	7.84	9	0.659	6	0.645	10	0.508	7	0.593	9
TFRC	8.44	10	0.712	13	0.541	3	0.525	10	0.644	13
RPL8	10.19	11	0.675	8	0.688	15	0.523	9	0.614	10
YWHAZ	10.22	12	0.705	12	0.681	14	0.566	13	0.284	5
RPL18	11.24	13	0.685	11	0.645	11	0.552	12	0.624	11
SDHA	12.40	14	0.760	15	0.615	7	0.591	15	0.672	15
LDHB	13.47	15	0.754	14	0.662	12	0.576	14	0.659	14
